# Cytochrome P450 Monooxygenase-Mediated Metabolic Utilization of Benzo[*a*]Pyrene by *Aspergillus* Species

**DOI:** 10.1128/mBio.00558-19

**Published:** 2019-05-28

**Authors:** Erin M. Ostrem Loss, Mi-Kyung Lee, Ming-Yueh Wu, Julia Martien, Wanping Chen, Daniel Amador-Noguez, Colin Jefcoate, Christina Remucal, Seunho Jung, Sun-Chang Kim, Jae-Hyuk Yu

**Affiliations:** a Molecular and Environmental Toxicology Center, University of Wisconsin—Madison, Madison, Wisconsin, USA; b Korea Research Institute of Bioscience and Biotechnology (KRIBB), Daejon, Republic of Korea; c Department of Bacteriology, University of Wisconsin—Madison, Madison, Wisconsin, USA; d College of Food Science and Technology, Huazhong Agricultural University, Wuhan, People’s Republic of China; e Department of Civil and Environmental Engineering, University of Wisconsin—Madison, Madison, Wisconsin, USA; f Department of Systems Biotechnology, Konkuk University, Seoul, Republic of Korea; g Department of Biological Sciences, Korea Advanced Institute of Science and Technology, Daejon, Republic of Korea; Karlsruhe Institute of Technology (KIT)

**Keywords:** *Aspergillus*, benzo[*a*]pyrene, catabolic enzyme system, cytochrome P450 monooxygenase, polycyclic aromatic hydrocarbons, *velvet* regulators, bioremediation, genome-wide expression, high-performance liquid chromatography, metabolomics, molecular genetics

## Abstract

Soil-dwelling fungal species possess the versatile metabolic capability to degrade complex organic compounds that are toxic to humans, yet the mechanisms they employ remain largely unknown. Benzo[*a*]pyrene (BaP) is a pervasive carcinogenic contaminant, posing a significant concern for human health. Here, we report that several *Aspergillus* species are capable of degrading BaP. Exposing Aspergillus nidulans cells to BaP results in transcriptomic and metabolic changes associated with cellular growth and energy generation, implying that the fungus utilizes BaP as a growth substrate. Importantly, we identify and characterize the conserved *bapA* gene encoding a cytochrome P450 monooxygenase that is necessary for the metabolic utilization of BaP in *Aspergillus*. We further demonstrate that the fungal NF-κB-type *velvet* regulators VeA and VelB are required for proper expression of *bapA* in response to nutrient limitation and BaP degradation in A. nidulans. Our study illuminates fundamental knowledge of fungal BaP metabolism and provides novel insights into enhancing bioremediation potential.

## Abstract (precis)

We are increasingly exposed to environmental pollutants, including the carcinogen benzo[*a*]pyrene (BaP), which has prompted extensive research into human metabolism of toxicants. However, little is known about metabolic mechanisms employed by fungi that are able to use some toxic pollutants as the substrates for growth, leaving innocuous by-products. This study systemically demonstrates that a common soil-dwelling fungus is able to use benzo[*a*]pyrene as food, which results in expression and metabolic changes associated with growth and energy generation. Importantly, this study reveals key components of the metabolic utilization of BaP, notably a cytochrome P450 monooxygenase and the fungal NF-κB-type transcriptional regulators. Our study advances fundamental knowledge of fungal BaP metabolism and provides novel insight into designing and implementing enhanced bioremediation strategies.

## INTRODUCTION

Polycyclic aromatic hydrocarbons (PAHs) are major soil pollutants that are formed by the partial combustion of organic matter and the five-ring PAH benzo[*a*]pyrene (BaP) poses a significant risk to human health (1). The increased use of hydrocarbons for energy during the past century has consequently increased the deposition of BaP, making it an abundant pollutant found in the environment (2).

**FIG 1:**
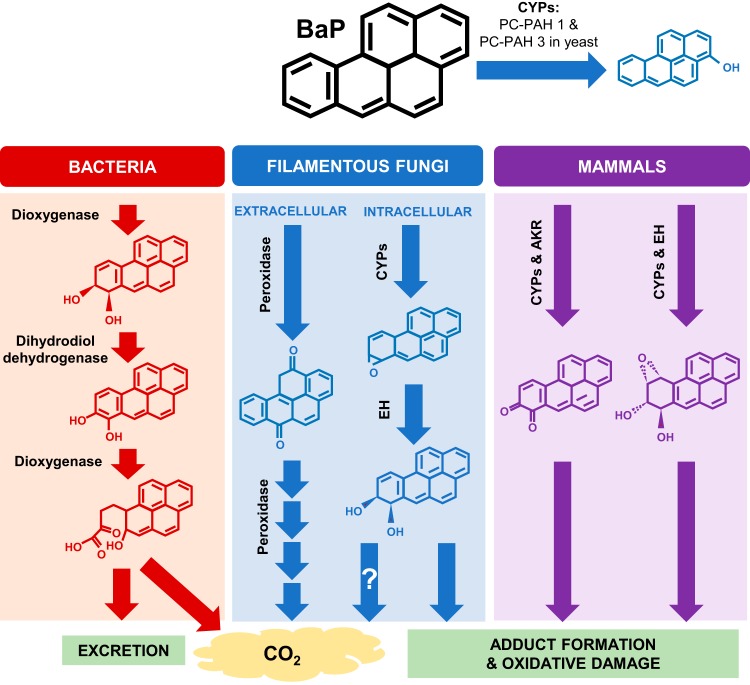
Schematic presentation of BaP transformation in mammals, fungi, and bacteria. Bacteria and fungi are both able to mineralize BaP; however, the initial enzymatic transformation steps differ drastically in that bacteria utilize dioxygenase enzymes, whereas fungi utilize extracellular peroxidase enzymes (46). Mammalian and intracellular fungal pathways overlap in their utilization of CYP enzymes yielding similar metabolites; however, far less is known about the specific CYPs and metabolites produced by fungi. The CYPs Pc-PAH1 and Pc-PAH3 in *P. chrysosporium*, which have the ability to convert BaP to 3-hydroxybenzo[*a*]pyrene when expressed in P. pastoris, are shown. Mammals utilize other enzymes in partnership with CYPs, such as epoxide hydrolases (EH) and aldo-keto reductases (AKR), but because the limited studies on fungal CYPs were done using heterologous expression, it is unknown whether other enzymes are involved in BaP metabolism and what the final metabolic products are.

Organisms have various ways of metabolizing BaP, depending on their ecological niche (Fig. 1). Saprophytic bacteria create BaP ring cleavage products, leading to usable nontoxic fragments (3). Humans are equipped with cytochrome P450 monooxygenases (CYPs) to transform and excrete BaP, but this process results in the creation of reactive intermediates, which cause adduct formation and oxidative stress in cells (4, 5). This makes BaP an especially harmful compound, resulting in cancer and immune dysregulation (2). In addition, its chemical properties make BaP stable in the environment and resistant to abiotic degradation (1).

Fungi are one of nature’s most resourceful organisms, accounting for up to 75% of the soil microbial biomass (6). *Aspergillus*, the most common genus of soil-dwelling fungi, frequently prevails in contaminated sites and can metabolize certain PAHs (7). *Aspergillus* species harbor abundant and diverse enzymatic systems, which allow them to metabolically utilize complex organic molecules that are highly toxic to animals (8, 9). However, specific genes involved in metabolic utilization of BaP in fungi remain to be revealed.

Part of the metabolic armory harbored by *Aspergillus* species is over 100 CYPs encoded in the genome (10). These enzymes participate in a variety of physiological activities that allow the fungi to adapt to new ecological niches. Soil is a hostile and competitive environment, so these CYPs play a role in the synthesis and degradation of various toxic compounds. Aspergillus nidulans contains 119 predicted CYPs, for which the functions of 13 have been determined experimentally, and 32 are positioned near key secondary metabolite synthases, suggesting their potential biosynthetic role (11). Therefore, a large number of CYPs have no known or predicted function.

The white rot fungus Phanerochaete chrysosporium has an outstanding capability for degrading and/or mineralizing high-molecular-weight PAHs and contains an extraordinarily large repertoire (over 150) of CYPs in its genome (12). An excellent study by Syed and colleagues identified and characterized six CYPs in *P. chrysosporium* (Pc-PAH1 to Pc-PAH6) capable of oxidizing different PAHs (13). These CYPs were inducible by naphthalene, phenanthrene, pyrene, and BaP. Expression of each of the six Pc-PAH CYPs in the yeast Pichia pastoris in conjunction with the homologous P450 oxidoreductase led to identification of Pc-PAH1 and Pc-PAH3 as CYPs with the ability to oxidize BaP to 3-hydroxybenzo[*a*]pyrene (13) (Fig. 1). This was the first report to identify a set of specific fungal CYPs having catalytic activity toward BaP. However, the functions of these CYPs have not been studied *in vivo* due to the limited ability of genetic manipulation in this organism, and hence further metabolism and the resulting products remain a mystery. Likewise, many reports about BaP-degrading fungal species isolated from contaminated sites lack systematic study due to limited genetic tools (7).

As *Aspergillus* species fill a similar saprophytic niche and have diverse metabolic capabilities, we hypothesize that they can metabolize BaP using a specific CYP-mediated pathway. We show that many, if not all, *Aspergillus* species can degrade BaP and uncover key aspects of cellular degradation of BaP by A. nidulans, using comprehensive genetic, genomic, and biochemical approaches. Importantly, we identify a gene (*bapA* [AN1884]) predicted to encode CYP617D1 and show that *bapA* is necessary for degradation of BaP *in vivo* in two *Aspergillus* species. These critical findings further allow us to investigate the *velvet* regulators associated with BaP metabolic degradation. Our study illuminates fundamental knowledge of fungal BaP metabolism and provides novel insight into designing and implementing enhanced bioremediation strategies.

## RESULTS

### *Aspergillus* species can degrade BaP effectively.

**FIG 2:**
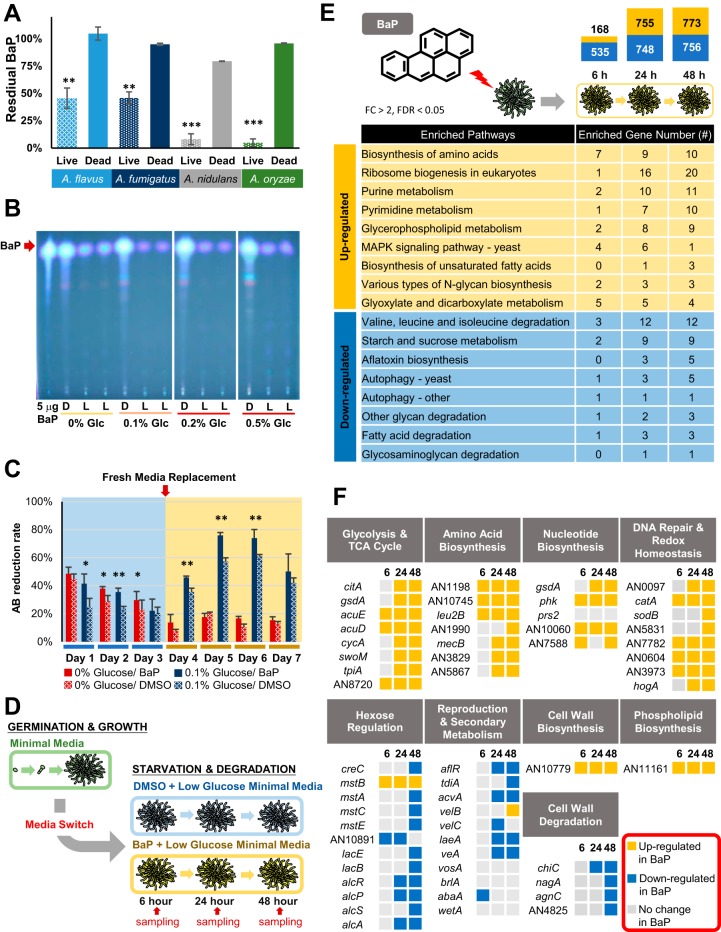
Degradation and utilization of BaP by *Aspergillus*. (A) Percentage of BaP remaining after 7-day cultures of each species. Three separate experiments were performed in triplicate. Mean values are plotted with standard errors of the mean (SEM) throughout all the graphs in the paper. **, *P* < 0.01; ***, *P* < 0.001. (B) Thin-layer chromatograms of residual BaP after 7-day cultures of A. nidulans in MM with various glucose concentrations. For reference, the 5-μg BaP standard is shown. Note the differences of the BaP intensity in dead cells (D) versus live cells (L). MM with 0.1% glucose with 200 μM BaP consistently resulted in the most effective degradation of BaP in multiple experiments. (C) alamarBlue reduction of A. nidulans cells treated with BaP and DMSO with (blue) or without (red) 0.1% glucose. Experiments were performed in triplicate. *, *P* < 0.05; **, *P* < 0.01. (D) Experimental overview for RNA sequencing. Samples were collected in triplicate at each time point. (E) KEGG pathways with upregulated (yellow) and downregulated (blue) DEGs at each time point. The bar chart indicates the total number of DEGs (log_2_ fold change [FC] of >1 or <−1 and *P* value of <0.05) at each time point. (F) Categories of transcriptional responses previously seen in carbon-starved A. nidulans cells and relative expression of specific genes in BaP- versus control (DMSO)-treated cells.

To test our initial hypothesis that *Aspergillus* species are able to degrade BaP, we employed A. nidulans, A. flavus, *A. oryzae*, and A. fumigatus. These fungal species are distantly related to each other, covering a broad range of *Aspergillus* (14). In all four species, the amount of BaP recovered from 7-day-old cultures was significantly lower in the living cells than that of dead cells (Fig. 2A), indicating that BaP was degraded or transformed in all species tested. The chromatogram showed no additional fluorescent peaks, suggesting that the degraded products were water soluble and/or not fluorescent at the same wavelengths as BaP (see Fig. S1 in the supplemental material). A. nidulans and *A. oryzae* were able to remove 92% ± 4.9% and 95% ± 3.5% of the added 200 μM BaP, respectively. As A. nidulans is a well-studied genetic model, we used it to further uncover the genetic and biochemical mechanisms of BaP degradation. Thin-layer chromatography (TLC) analyses of residual BaP in different concentrations of glucose in minimal medium (MM) have revealed that MM with 0.1% glucose led to the most effective degradation of BaP by A. nidulans (Fig. 2B).

10.1128/mBio.00558-19.1 FIG S1 Chromatogram showing compounds extracted from whole-cell WT cultures. Extract from A. nidulans cultures after 7 days of incubation with 200 μM BaP was prepared and analyzed by HPLC as described in Materials and Methods. Pyrene (4-ring molecule at ∼6.2 min) and BaP peak retention times matched those of authentic standards, as indicated by chemical structure. No additional peaks other than the extracted PAHs were detected. Download FIG S1, PDF file, 0.1 MB. Copyright © 2019 Ostrem Loss et al. 2019 Ostrem Loss et al. This content is distributed under the terms of the Creative Commons Attribution 4.0 International license.

### BaP increases fungal cell viability.

To test whether A. nidulans uses BaP as a substrate for growth, we measured changes in cell viability. The alamarBlue assay showed that cells exposed to BaP had significantly higher viability than the dimethyl sulfoxide (DMSO) controls, including time points after the addition of fresh medium to supply cells with the additional nutrients for the continuous proliferation (Fig. 2C). As found in TLC data, the prolonged viability of the cells with BaP was observable with the presence of a small amount of glucose in the medium (0.1%), whereas BaP addition alone was able to increase the cell viability at 2 days postexposure (Fig. 2C). This suggests that BaP can be used as a carbon source, and such effects can be enhanced with the supplementation of a small amount of glucose, which may provide additional resources for energy and enzyme production.

### BaP leads to upregulation of cell growth-associated genes.

To further test the hypothesis that the fungus uses BaP as a growth substrate, we investigated the genome-wide expression responses of A. nidulans to BaP via transcriptome sequencing (RNA-seq). Transcript levels were measured in BaP-treated cells relative to controls (DMSO) at 6, 24, and 48 h postexposure by KEGG analysis (Fig. 2D). The number of differentially expressed genes (DEGs) was low at 6 h (703 DEGs); this increased to 1,503 and 1,529 DEGs at 24 and 48 h, respectively (Fig. 2E). KEGG pathway analysis indicated that, as time progresses with BaP exposure, genes associated with cell growth, such as ribosome biogenesis, biosynthesis of amino acids, nucleotide metabolism, biosynthesis of unsaturated fatty acids, *N*-glycan biosynthesis (cell wall), and glycerophospholipid (membrane), were upregulated (Fig. 2E). Conversely, genes categorized into the pathways indicative of cell starvation and stress, including amino acid degradation, autophagy, aflatoxin (sterigmatocystin) biosynthesis, and starch metabolism, were downregulated in BaP-treated cells (Fig. 2E). These results indicate that BaP-treated cells are actively growing compared to controls and that the fungus is able to use BaP to sustain growth.

The transcriptomic response to carbon starvation in A. nidulans shows upregulation of genes involved in programmed cell death, macroautophagy, cell wall component degradation, asexual reproduction, and secondary metabolite production (15). Downregulation of genes involved in glycolysis and oxidative phosphorylation, cell wall component synthesis, and nitrogen and lipid anabolic pathways was also seen in the starving cells (15). We carried out an integrated analysis of the differentially expressed genes by BaP treatment with the carbon starvation stress response and found that the BaP-treated cells showed upregulation of the following genes: (i) *citA*, *gsdA*, *acuE*, *acuD*, and *cycA*, involved in the tricarboxylic acid (TCA) cycle, replenishment of TCA cycle intermediates, and oxidative phosphorylation; (ii) AN11161, involved in phospholipid biosynthesis; (iii) AN10779, involved in β-1,6-glucan biosynthesis; and (iv) several genes involved in amino acid biosynthesis pathways (Fig. 2F). BaP-treated cells show downregulation of *aflR* and other sterigmatocystin biosynthesis genes, *abaA*, involved in conidiation, and *agnC*, *nagA*, *chiC*, and AN4825, involved in cell wall component hydrolase enzymes (Fig. 2F). Taken together, the data demonstrate that BaP enables the cells to grow more actively than control cells and provide some evidence that BaP is metabolized and shuttled into carbon utilization pathways.

Finally, in an attempt to address whether BaP metabolism causes oxidative stress and/or DNA damage responses in A. nidulans as in mammalian cells, we compared our RNA-seq data with those representing responses to cells treated with other known oxidizing compounds. Some redox-balancing genes were upregulated in BaP-treated cells, including *catA* and *sodB* (Fig. 2F). Expression of some DNA repair genes was also induced by BaP, including AN0604 and AN0097 (Fig. 2F). These results suggest that BaP may cause DNA damage in A. nidulans as in mammalian cells, although it is shown that fungi have additional capacity to prevent extensive DNA damage (16).

### Identification of CYP necessary for metabolic utilization of BaP.

Initial oxidation of BaP in mammalian cells is mediated by a CYP, adding a single molecular oxygen, leading to the formation of BaP epoxide intermediates, which can be further converted into hydroxylated products (4, 17–19).

**FIG 3:**
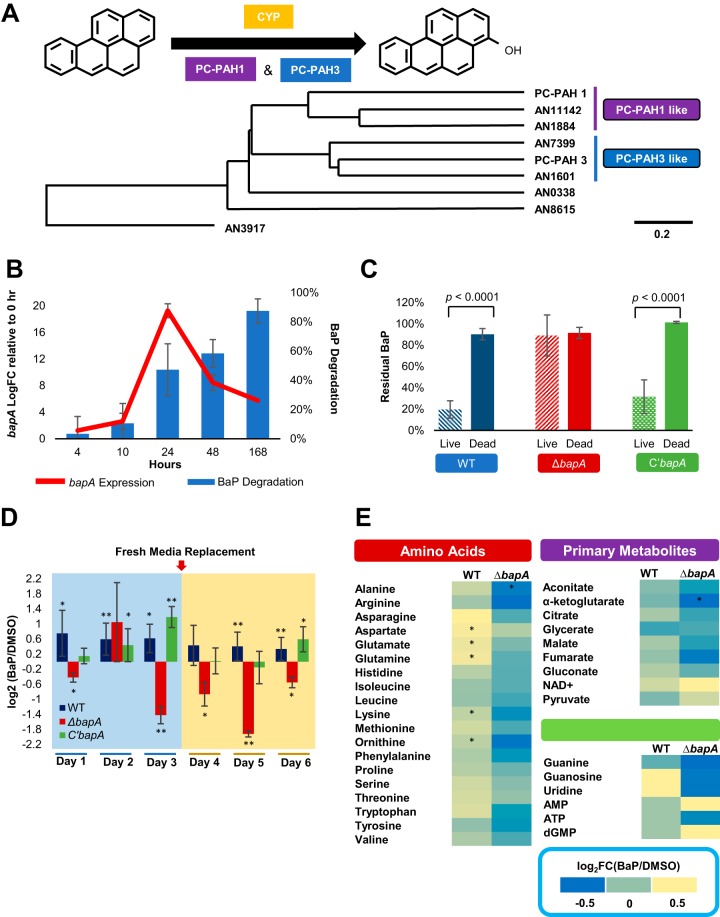
Identification and characterization of the CYP BapA (AN1884) necessary for metabolic utilization of BaP. (A) Phylogenetic tree representing A. nidulans CYPs similar to Pc-PAH1 and Pc-PAH3. (B) Correlation of *bapA* (AN1884) mRNA levels and BaP degradation. Time point 0 indicates exponential growth (18 h postgermination) in regular MM (1% glucose), and each time point represents hours after the switch to 0.1% glucose MM. The mean is plotted as a bar, and error bars represent standard error. BaP degradation (blue bar) was determined by measuring residual BaP compared to the initial 200 μM BaP. (C) Residual BaP in each indicated strain. Three separate experiments were performed in triplicate. Note the lack of BaP degradation in the Δ*bapA* mutant compared to WT and complemented (C′) strains (*P* < 0.001). *, *P* < 0.05; **, *P* < 0.01; and ***, *P* < 0.001. (D) Comparison of the log_2_ values of the alamarBlue reduction levels in WT, Δ*bapA*, and C′ strains in cells treated with BaP relative to DMSO only (DMSO) at days 1 to ∼6. “Fresh media replacement” indicates collection and washing of cells and addition of fresh medium. All treatments were performed in triplicate, and Student's *t* test was used (*, *P* < 0.05; **, *P* < 0.01). (E) Relative quantification of cellular components in the WT and the Δ*bapA* mutant. Each colored square represents log_2_ fold change (FC) of the mean quantity of the metabolites in BaP versus DMSO. Each sample was prepared using biological triplicates. *, *P* < 0.05.

With the hypothesis that A. nidulans employs a CYP to degrade BaP, we first examined our RNA-seq data to search for the CYP genes upregulated by BaP treatment and found that no specific CYPs were clearly induced by BaP. We then used the CYPs of *P. chrysosporium*, Pc-PAH1 and Pc-PAH3, which when expressed in the yeast cells, converted BaP to 3-hydroxybenzo[*a*]pyrene (13), to search for closely related CYPs in the A. nidulans genome. Despite the 723 million years of divergence between the two fungi (20), several CYPs similar to Pc-PAH1and Pc-PAH3 were identified (Fig. 3A; see Table S1 in the supplemental material). Among these, mRNA levels of AN1884 in glucose-limited medium and kinetics of BaP degradation were closely aligned, indicating that this CYP may be associated with metabolism of BaP (Fig. 3B; Fig. S2B).

10.1128/mBio.00558-19.2 FIG S2 Levels of *bapA* mRNA increase as glucose becomes limited. (A) Levels of *bapA* (AN1884) mRNA in MM with various concentrations of glucose. Levels were quantified using 2^−ΔΔ^*^CT^*, in which *bapA* expression (*C_T_*) was found relative to the reference gene *actA* (γ-actin) (Δ*C_T_*) and then relative to time point 0 (ΔΔ*C_T_*). Time point 0 indicates exponential growth (18 h postinoculation) in MM with glucose, and each time point represents hours after the switch to MM with the indicated glucose concentration (0% to ∼10%). Complete medium is indicated by “2%+.” Each measurement was performed using technical triplicates for RT-qPCR accuracy, and biological triplicates were used for each time point. The mean is plotted as a bar, and the error bars represent standard deviation. (B) Correlation of levels of *bapA* (AN1884) mRNA and BaP degradation (shown in Fig. 3B) presented in a scatter plot. (See the legend to Fig. 3B.) Linear regression was determined using only early time points, and the *R*^2^ value was calculated using only the first three points. Download FIG S2, PDF file, 0.2 MB. Copyright © 2019 Ostrem Loss et al. 2019 Ostrem Loss et al. This content is distributed under the terms of the Creative Commons Attribution 4.0 International license.

10.1128/mBio.00558-19.6 TABLE S1 Oligonucleotides used in this study. Download Table S1, DOCX file, 0.01 MB. Copyright © 2019 Ostrem Loss et al. 2019 Ostrem Loss et al. This content is distributed under the terms of the Creative Commons Attribution 4.0 International license.

To test its function in BaP metabolism *in vivo*, we generated independent deletion (Δ) strains of AN1884 in A. nidulans and found that all three independent null mutant strains lost the ability to degrade BaP, with no distinct growth and developmental phenotypic changes (Fig. S3). Reintroducing an AN1884 coding region into null mutant strains restored the BaP degradation ability to that of the wild type (WT) (Fig. 3C), supporting the hypothesis that AN1884 is responsible for the breakdown of BaP, so it was named *bapA* (benzo[a]pyrene metabolism locus A). Measuring the cell viability of the WT, the Δ*bapA* mutant cells, and complemented (C′*bapA*) cells treated with BaP relative to those treated with DMSO revealed that the Δ*bapA* mutant cells were not only less viable than WT and C′*bapA* cells treated with BaP, but less viable than Δ*bapA* mutant cells treated with DMSO (Fig. 3D). This indicates BaP may be toxic to cells unable to metabolize it. To our knowledge, this is the first report providing evidence of *in vivo* function of a CYP in cellular degradation of BaP.

10.1128/mBio.00558-19.3 FIG S3 The deletion of *bapA* abolishes the BaP degradation ability without affecting growth and development in A. nidulans. (A) Colony photographs of WT (rJMP1.59) and two independent Δ*bapA* strains (no. 35 and 47) taken at 3 days post-point inoculation at the center of solid MM plus glucose. (B and C) Thin-layer chromatograms (B) and a bar graph (C) showing the residual BaP upon 7 days of culture of the WT and three independent Δ*bapA* strains (no. 1, 35, and 47) (see Materials and Methods). Statistical significance was determined by Student’s *t* test (***, *P* < 0.001). Download FIG S3, PDF file, 0.10 MB. Copyright © 2019 Ostrem Loss et al. 2019 Ostrem Loss et al. This content is distributed under the terms of the Creative Commons Attribution 4.0 International license.

We attempted to identify the BaP metabolite(s) formed by BapA. We isolated microsome-containing fractions from the WT, Δ*bapA*, and C′*bapA*, cells and incubated them with BaP. The WT and C′*bapA*, but not Δ*bapA*, chromatograms showed a small fluorescent peak with a shorter retention time than BaP (Fig. S4A). The retention time of this unknown metabolite was compared to those of known BaP metabolites and matched that of benzo[*a*]pyrene-3,6-dione (Fig. S4A). However, the fluorescence spectra revealed that sample peaks do not show the same fluorescing as benzo[*a*]pyrene-3,6-dione (Fig. S4B). No other known metabolite standards matched the retention time of this peak (Fig. S4C). Although we were not able to reveal the exact identity of the product formed within the microsome fraction of cells expressing BapA, we were able to rule out many BaP metabolites formed by other organisms. This may indicate that BapA is involved in forming a unique metabolite not previously reported in other organisms.

10.1128/mBio.00558-19.4 FIG S4 Microsome analyses and related information. (A) Chromatogram of compounds extracted from BaP incubated with the microsome fraction of each strain indicated or authentic standard. Peaks with a retention time around 3.2 min are enlarged to show differences in the WT, Δ*bapA*, and complemented *bapA* strains (blue box). Benzo[*a*]pyrene recovery was quantified by normalizing with the recovered internal standard pyrene. The mean of two replicated experimental data is plotted as a bar, and error bars represent standard error. (B) Fluorescence spectra collected at the apex of the peak with a retention time of 3.2 min and authentic standard of benzo[*a*]pyrene-3,6-dione. (C) Chromatogram of authentic standards of known BaP metabolites. Metabolite standards (from left to right) benzo[*a*]pyrene-7,8-diol, benzo[*a*]pyrene-3,6-dione, and benzo[*a*]pyrene-7,8-dione, pyrene, and BaP, as indicated by chemical structure, were diluted to 1 μM in 1:1 solvent A-solvent B and run by the HPLC method described in Materials and Methods with FLD λ_excitation_ of 248 nm and λ_emission_ of 465 nm. Download FIG S4, PDF file, 0.2 MB. Copyright © 2019 Ostrem Loss et al. 2019 Ostrem Loss et al. This content is distributed under the terms of the Creative Commons Attribution 4.0 International license.

The availability of the Δ*bapA* mutant allows us to further investigate the downstream metabolomic outcomes of BaP. Since BaP treatment caused upregulation of genes associated with amino acid biosynthesis, which is an easily measured endpoint for carbon utilization, we quantified free amino acids in WT and Δ*bapA* cells treated with BaP relative to the control (DMSO). In agreement with alamarBlue data, only BaP-treated WT cells showed significant accumulation of glutamate, aspartate, glutamine, lysine, and the intermediate amino acid ornithine (Fig. 3E). The Δ*bapA* cells showed significant accumulation of alanine and α-ketoglutarate in control (DMSO) compared to BaP treatment (Fig. 3E). This provides additional evidence that loss of BaP metabolism causes lack of cell growth upon exposure to BaP and alludes to toxicity caused by the lack of BaP-degrading ability. While insignificant due to large deviations among samples, accumulation of several primary metabolites involved in energy metabolism, such as citrate and malate, and nucleotides involved DNA/RNA synthesis or signaling, such as cyclic AMP (cAMP), appeared to be affected by BaP (Fig. 3E).

### BapA is widely distributed in ascomycota and is functionally conserved in A. flavus.

**FIG 4:**
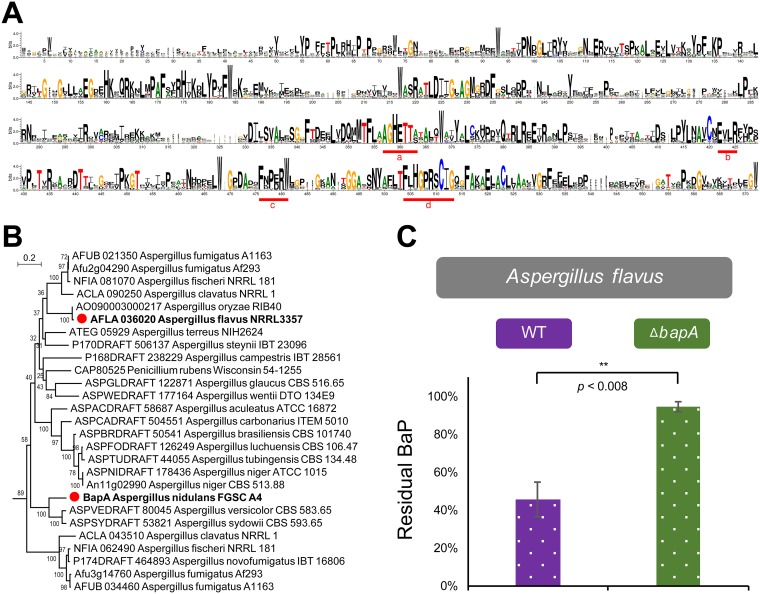
The BapA CYP signature motifs, distribution of BapA in *Aspergillus* species, and the role of BapA in BaP degradation in A. flavus. (A) Conserved domains of the CYP617 family proteins queried from FungiDB (42) showing greater than 40% identity against the A. nidulans BapA protein (listed in Fig. S5). (B) Phylogenetic tree of the CYP617D members in *Aspergillus* having greater than 55% identity against the A. nidulans BapA protein. These proteins can be classified into the same subfamily, CYP617D, based on the rules of the International P450 Nomenclature Committee. For the expanded information, see Fig. S5 and Table S3. (C) Residual BaP in WT and the Δ*bapA* mutant of A. flavus. All experiments were performed in triplicate, and three independent Δ*bapA* strains were tested. **, *P* = 0.008.

The structural analysis showed that the four widely recognized consensus regions (a to d) (Fig. 4A) contributing to the core function of P450s (10) are highly conserved in the CYP617 family. Interestingly, the conserved motif a (AGHETT) of the CYP617 family is very specific and is highly similar to those of archaea and bacteria (10).

10.1128/mBio.00558-19.5 FIG S5 Phylogenetic tree showing all fungal proteins with greater than 40% sequence identity to A. nidulans BapA (marked by a red dot). *Aspergillus* CYPs showing greater than 55% sequence identity to BapA (shown in Fig. 4B) are marked by red brackets. The A. nidulans CYP51 protein (AN8283) was used as the out-group. Download FIG S5, PDF file, 0.3 MB. Copyright © 2019 Ostrem Loss et al. 2019 Ostrem Loss et al. This content is distributed under the terms of the Creative Commons Attribution 4.0 International license.

10.1128/mBio.00558-19.7 TABLE S2 CYPs similar to Pc-PAH1 and Pc-PAH3 in A. nidulans, A. flavus, and A. fumigatus. Download Table S2, DOCX file, 0.1 MB. Copyright © 2019 Ostrem Loss et al. 2019 Ostrem Loss et al. This content is distributed under the terms of the Creative Commons Attribution 4.0 International license.

10.1128/mBio.00558-19.8 TABLE S3 Gene number distribution of the CYP617 family members in the kingdom Fungi and oomycetes. Download Table S3, XLSX file, 0.1 MB. Copyright © 2019 Ostrem Loss et al. 2019 Ostrem Loss et al. This content is distributed under the terms of the Creative Commons Attribution 4.0 International license.

We performed a phylogenic analysis to determine how widely distributed BapA is in fungi. We found 64 CYP617 family members in the fungal kingdom, covering dothideomycetes, eurotiomycetes, leotiomycetes, and sordariomycetes, with the number ranging from one to several per species (see Fig. S5 and Table S3 in the supplemental material). CYP617 members are limited to ascomycetes. The BapA subfamily CYP617D1 was mostly distributed in the genus *Aspergillus* (Fig. 4B), suggesting a conserved role within the genus.

To examine a potential conservation of its function, we identified a likely orthologue of BapA in A. flavus (AFLA_036020) and generated three individual null mutant strains. All Δ*AFLA_036020* strains lost the ability to degrade BaP, corroborating the conserved and essential role of BapA in degradation of BaP in *Aspergillus* species (Fig. 4C) under glucose-limiting conditions.

### Requirement of fungal NF-κB-type regulators in BaP degradation.

Identification of *bapA* allowed us to further investigate its upstream regulatory components. The *velvet* proteins are a family of global transcription factors (TFs) involved in diverse aspects of fungal biology (21). They contain a DNA binding domain structurally similar to that of the human TF complex nuclear factor kappaB (NF-κB) (22) p50, which governs cell survival upon exposure to BaP (23–26).

**FIG 5:**
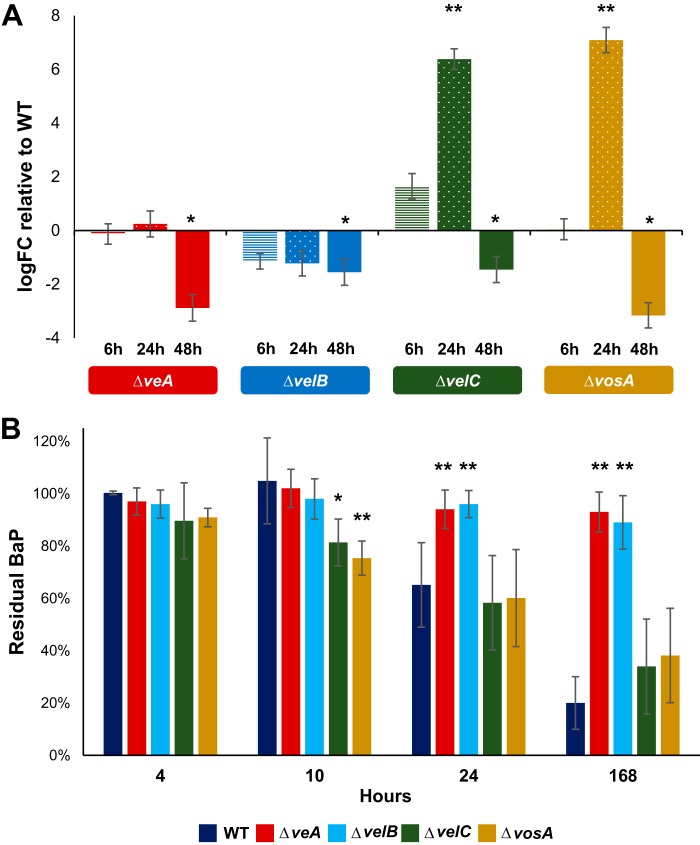
Fungal NF-κB-type *velvet* regulators are required for proper *bapA* expression and BaP degradation. (A) Levels of *bapA* mRNA were measured using 2^−ΔΔ^*^CT^* relative to *actA* (γ-actin) (Δ*C_T_*) and then relative to the WT (ΔΔ*C_T_*). Time points indicate hours after the switch to 0.1% glucose medium. Technical and biological triplicates were used for each time point and treatment. *, *P* < 0.05; **, *P* < 0.01. (B) Residual BaP remaining after being cultured for 7 days with each strain. Note the lack of BaP degradation in Δ*veA* and Δ*velB* strains. Three separate experiments were performed in triplicate. *, *P* < 0.05; **, *P* < 0.01.

Due to this structural similarity and diverse regulatory functions between human NF-κB and the fungal *velvet* complex, we hypothesized that *velvet* proteins might play a role in BaP degradation. To address this hypothesis, we first tested mRNA levels of *bapA* in each *velvet* deletion mutant (Δ*veA*, Δ*velB*, Δ*velC*, and Δ*vosA*) and found that the two regulators VeA and VelB were necessary for proper expression of *bapA* (Fig. 5A). Interestingly, the other two regulators, VosA and VelC, seem to play a repressive role at 10 h post-glucose starvation and an activating role at later time points (Fig. 5A). To verify that expression of *bapA* translates into BaP degradation, we tested the degradation ability of each deletion mutant and found that the Δ*veA* and Δ*velB* mutants were unable to degrade BaP. On the contrary, the Δ*vosA* and Δ*velC* mutants were able to degrade the same amount of BaP, yet faster than the WT (Fig. 5B). These results indicate that VeA and VelB play a key role in metabolic utilization of BaP, in part by controlling proper expression of *bapA*, whereas VosA and VelC may indirectly play a role in coordinating the proper timing of BapA expression in response to glucose limitation.

## DISCUSSION

BaP is a contaminant of significant concern because of its ubiquity and toxicity. As a result of its stability, biologically driven degradation remains the predominant form of removal from the environment (27). Thus, understanding how saprophytic bacteria and fungi effectively metabolize BaP is critical for the effective removal of BaP.

**FIG 6:**
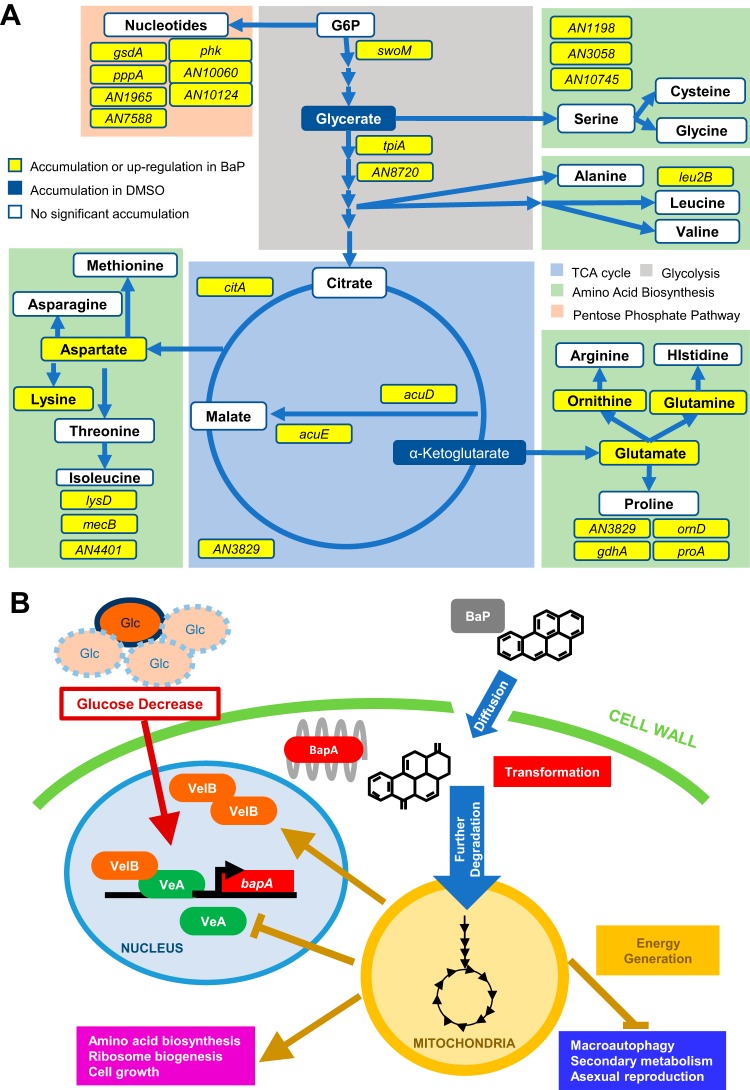
Models for BaP metabolic utilization in A. nidulans. (A) BaP confers changes in energy generation pathways indicated by colored blocks. Upregulated DEGs are shown in yellow. Amino acids and other compounds found in greater quantity as a result of BaP treatment in WT but not Δ*bapA* strains are shown in yellow and white boxes, where yellow indicates statistical significance (*P* < 0.05). Blue boxes represent cellular components that significantly decrease as a result of BaP treatment in WT but not in Δ*bapA* strains (*P* < 0.05). (B) Proposed genetic pathway for the metabolic utilization of BaP in A. nidulans. Limited glucose and the VeA-VelB complex are associated with increased expression of *bapA*.

This is the first comprehensive study showing that *Aspergillus* species can effectively degrade BaP, resulting in cell survival and growth during carbon starvation (Fig. 6A). We were unable to identify specific BaP intermediates in this study, so it is unclear which pathways are involved in further metabolism of BaP. The CYP-mediated metabolism of BaP in human cells has been well characterized, so we attempted to use BaP metabolite standards to identify the potential metabolite peak using high-performance liquid chromatography (HPLC). None of the standards we tried matched the retention time or absorbance spectrum of the peak. Additionally, CYP metabolism of BaP in mammalian cells causes mutagenic and cytotoxic effects (28), whereas we observed an increase in viability of A. nidulans cells exposed to BaP. Together this leads us to conclude that BaP metabolism in *Aspergillus* sp. involves the unique CYP BapA, and further degradation of BaP may occur via metabolic pathways not found in mammalian cells. Further study is needed to understand the full metabolic pathway(s) of BaP degradation.

Our study does, however, identify a necessary CYP617D1 enzyme that not only provides information that can help effectively implement bioremediation strategies, but also gives us a unique insight into evolution of the fungal CYPs and their biocatalytic activity. We propose a model in which VeA and VelB activate expression of *bapA* in response to nutrient limitation and BapA oxidizes BaP (Fig. 6B). We hypothesize that oxidized BaP is further enzymatically fragmented, and the carbon is shuttled into energy-generating pathways, which in turn represses further expression of *bapA* (Fig. 6B).

Filamentous fungi harbor many more CYPs relative to their genome size than animals and bacteria, yet the functions of many remain unknown. The diversity of CYPs in fungi could be due to their need to metabolize many different carbon sources found in soils, including large cyclic compounds like lignin and plant polymers. It is also feasible that fungi, like animals, may need detoxification systems reliant on CYP activity to avoid toxic compounds produced by competing microbes and plants. Our results demonstrate that the regulation of *bapA* is governed by response to carbon starvation, rather than exposure to the toxicant BaP.

The A. nidulans, A. flavus, and A. fumigatus genomes each contain over 100 encoded CYPs, with 90, 93, and 57 family types, respectively, yet only 45 types are shared (10). Despite this diversity, BapA (CYP617D1) is found in all three distantly related *Aspergillus* and *Penicillium* species, and in A. nidulans and A. flavus it plays the same functional role of degrading BaP. Because CYPs demonstrate substrate promiscuity, it is likely that BapA oxidizes other compounds, such as other PAHs and/or large planar endogenous compounds. The deletion of *bapA* showed no obvious growth and developmental changes, suggesting that BapA does not likely play a major housekeeping role.

Regulation of *bapA* also demonstrates a novel understanding of how *Aspergillus* species respond to organic contaminants like BaP. Humans and fungi have evolved different strategies to deal with exposure to xenobiotics, yet both employ CYPs. Humans do not invest energy into utilizing carbon sources more complex than various sugars and a few of their polymers, so CYP transformation of BaP yields more polar metabolites that can then be excreted. Regulation of encoded BaP-metabolizing CYPs is predominantly governed by the aryl hydrocarbon receptor (29), yet BaP and its metabolites also activate NF-κB (23–25). NF-κB is a protein heterodimer consisting of p50 and RelA, which upon activation by many types of cellular stress, from microbial and viral proteins to ionizing radiation, promotes cell survival (26). Filamentous fungi, on the other hand, act more as ecological scavengers and are capable of utilizing large carbon-containing compounds, such as plant cell wall polymers. These fungi have evolved with the global regulators called the *velvet* proteins with a DNA binding domain structurally similar to that of NF-κB p50 (22). The *velvet* regulators in *Aspergillus* species govern environmental sensing, orchestration of cell growth, reproduction, stress response, spore viability, and biosynthesis of various secondary metabolites, which similarly helps the fungal cells to survive environmental stressors (21, 30).

In this study, we have shown that CYP-mediated degradation of BaP requires functions of the *velvet* family proteins VeA and VelB. These regulatory proteins control expression of *bapA* in response to stress resulting from carbon insufficiency, as opposed to exposure to xenobiotics. As this CYP is functionally conserved across distantly related fungi, it may play the same role in many ascomycete fungi. Further investigation of substrates metabolized by BapA would reveal its activity on other environmental contaminants as well as give insight into a possible endogenous function.

## MATERIALS AND METHODS

### Strains, media, and culture conditions.

The *Aspergillus* strains used in this study are listed in Table 1. Initially, 10^6^ spores/ml were added to 400 ml minimal medium (MM) (31) with 1% glucose in 2-liter flasks and incubated for 18 h at 220 rpm at 37°C. The mycelial aggregates were then collected on sterile Miracloth (Sigma-Aldrich), rinsed, and transferred to 100 ml MM with 0.1% glucose in 250-ml Erlenmeyer flasks. Control dead cells were autoclaved on a liquid cycle at 121°C for 20 min to account for any nonmetabolic sources of loss of BaP. A 100 mM stock solution of BaP (Sigma-Aldrich) in dimethyl sulfoxide (DMSO) was added to the cultures to a final concentration of 200 μM; the same volume of DMSO was added to controls. All flasks were further incubated at 220 rpm at 37°C for the designated time. Escherichia coli DH5α cells were grown in Luria-Bertani medium with ampicillin (100 μg/ml) for plasmid amplification.

**TABLE 1:** *Aspergillus* strains used in this study

Strain	Genotype	Source or reference^*a*^
A. nidulans FGSC4	WT, *veA^+^*	FGSC
A. flavus NRRL 3357	WT	FGSC
A. flavus 3357.5	*pyrG^−^*	47
A. fumigatus AF293	WT	48
*A. oryzae* M2040	WT	KACC
A. nidulans RJMP1.59	*pyrG89 pyroA4 veA*^+^	49
A. nidulans TMK6	*pyrG89 pyroA4* Δ*AnibapA*::*AfupyrG*^+^ *veA*^+^	This study
A. flavus TEO1	Δ*AflbapA*::*AfupyrG^+^ pyrG^−^*	This study
A. nidulans TEO2	*pyrG89 pyroA*::*bapA*(*p*)::*bapA*::FLAG_3×_::*pyroA*^*b*^ Δ*bapA*::*AfupyrG^+^ veA^+^*	This study
A. nidulans THS15	*pyrG89 pyroA4* Δ*vosA*::*AfupyrG*^+^ *veA*^+^	44
A. nidulans THS16	*pyrG89 pyroA4* Δ*velB*::*AfupyrG*^+^ *veA*^+^	44
A. nidulans THS11	*pyrG89 pyroA4* Δ*velC*::*AfupyrG*^+^ *veA*^+^	50
A. nidulans THS17	*pyrG89 pyroA4 ΔveA*::*AfupyrG^+^ veA^+^*	44

a FGSC, Fungal Genetic Stock Center; KACC, Korean Agricultural Culture Collection.

b The 3/4 *pyroA* marker causes targeted integration at the *pyroA* locus.

### Extraction and HPLC analysis.

Extraction of BaP was optimized to recover all BaP adhered to and taken up by cells, but not biotransformed. Individual fungal cell cultures were extracted using 100 ml 1:1 hexane-ethyl acetate with pyrene (Sigma-Aldrich [final concentration, 200 μM]) as an internal standard to correct for extraction efficiency. The entire mixture was sonicated using a Sonic dismembrator model 100 (Fisher Scientific) with a 1/2-in. probe on full power for 6 min to ensure disintegration of hyphal pellets. Solvent (1 ml) was removed and centrifuged to remove particulate matter and diluted 100-fold in 1:1 solvent A (30 mM acetate buffer at pH 4.7, 10% acetonitrile)-solvent B (acetonitrile). BaP and pyrene were quantified by high-performance liquid chromatography (HPLC) using an Agilent 1260 system equipped with a 3- by 50-mm Poroshell 120 EC-C_18_ 2.7-μm column (Fig. S1). A linear gradient that ramped from 55% B to 90% B over 10 min at a flow rate of 0.75 ml/min was used, followed by fluorescence detection (FLD [λ_excitation_ = 248 nm and λ_emission_ = 465 nm]). All standard curves were linear, and the detection limits were ≤0.1 μM for pyrene and BaP.

### TLC analysis of residual BaP.

To further verify the degradation of BaP by A. nidulans at different glucose concentrations (Fig. 2B), we also carried out TLC analyses 10 times and obtained a high degree of reproducibility. BaP extraction was performed by adding 0.5% (vol/vol) 6 N HCl (to stop all metabolic activity) to the fungal cultures (100 ml). The mycelium was collected through Miracloth and squeezed to maximize collection of the supernatant, which was transferred to a fresh 250-ml flask and mixed with 100 ml of ethyl acetate (1:1 ratio). Both liquids were then transferred to a new 250-ml separatory funnel. After shaking vigorously for 2 min, the organic phase was transferred in a new flask. Fresh solvent was added to the separatory funnel, and shaking and collecting were repeated two additional times. The resulting solvent was allowed to evaporate in the fume hood, and each dried sample was resuspended with ethyl acetate (1 ml) for TLC analysis. Ten microliters of each sample was applied to a TLC silica plate, including a fluorescence indicator (Kiesel gel 60, 0.25 mm thick; Merck). Authentic BaP standard was loaded as a control. The TLC plate was then developed with toluene-acetone-hexane (1:1:1 [vol/vol/vol]), where the *R_f_* value of BaP was 0.9. The TLC plate was exposed to UV at *A*_320_ for 30 s, and images were captured using a Canon EOS camera. To quantify the residual BaP shown in Fig. S3B, the density of each BaP spot on TLC was determined using ImageJ (NIH): the relative amount of BaP in live cells to the dead cell control (=100%) is presented.

### alamarBlue reduction assay.

Cell viability was determined by percentage of alamarBlue (Bio-Rad) reduction as described previously (32), with the following exceptions. Cells were prepared as described for BaP degradation with solvent (DMSO) only as a control, and 0.45 ml of cells was added to 0.45 ml fresh MM with 0.1% glucose and 100 μl alamarBlue and incubated for 2 h at 37°C.

### RNA preparation and qRT-PCR.

Fungal cells from submerged cultures were collected at designated time points, squeeze-dried, flash frozen in liquid N_2_, and stored at −80°C until subjected to RNA preparation. Total RNA isolation was done using TRIzol as described previously (33). cDNA was prepared using an avian myeloblastosis virus (AMV) reverse transcriptase kit (NewEngland Biolabs) with oligo(dT). Reverse transcriptase quantitative PCR (RT-qPCR) was performed with iTaq universal SYBR green supermix (Bio-Rad) on a Bio-Rad CFX96 real-time PCR detection system. mRNA was normalized using threshold cycle (2^−ΔΔ^*^CT^*) method (34). Levels of *bapA* mRNA were determined using 2^−ΔΔ^*^CT^*, in which *bapA* expression (*C_T_*) was found relative to the reference gene *actA* (γ-actin) (Δ*C_T_*) and then relative to time point 0 (ΔΔ*C_T_*). Time point 0 indicates exponential growth (18 h postgermination) in regular MM (1% glucose), and each time point represents hours after the switch to MM with 0.1% glucose. Each experiment was performed using technical triplicates for RT-qPCR accuracy, and three biological triplicates were used for each time point. The oligonucleotides used are listed in Table S1. Total RNA was extracted and submitted to ProteinCT Biotechnologies (Madison, WI) for library preparation and RNA sequencing.

### RNA sequencing.

RNA sequencing was done as described previously (35). The library was constructed and purified and sequenced (SE100bp) using the Illumina HiSeq2500, and over 20 million high-quality reads per sample were achieved.

### Data QC and analysis.

Verification of the quality of reads (quality control [QC]), alignments, gene annotation, and differential expression analysis were performed as described previously (35).

### Functional enrichment analysis (KEGG).

The KEGG pathway database was used to search against A. nidulans KEGG pathway maps in order to identify A. nidulans metabolic pathways with the differentially expressed genes (DEGs) after exposure to BaP on 20 February 2018 (36).

### Metabolomics of amino acid and primary metabolites.

Fungal cells prepared as described for BaP degradation with the DMSO control were subject to extraction of cellular components as described previously (37), with the following exceptions. Hyphal mats were filtered and squeeze-dried, noting the mass after removing liquid, 1 day after transfer to BaP-containing medium. Tissue was flash frozen in liquid N_2_ and stored at −80°C. Two milliliters of extraction solvent (37) was added, and samples were sonicated using a 1/4-in. probe for 3 min and centrifuged to remove cell debris. Additional sample prep and analysis were performed as described previously (37).

### Protein alignment.

CYP sequences similar to those of Pc-PAH1 and Pc-PAH3 in *Aspergillus* sp. were identified using blastp (38). Protein sequences were found using NCBI, and protein alignment was calculated using Clustal Omega at EMBL-EBI output ClustalW with character counts (39). A phylogenetic tree was created using Jalview nearest neighbor joining (40).

### Analysis of BapA families.

BapA (AN1884) was assigned to CYP617D1 (11) and analyzed according to the rules of the International P450 Nomenclature Committee (41). BapA protein sequence was used to query FungiDB (42). CYP617 members were aligned, and the phylogenetic tree was constructed as previously described (43).

### Generation of Δ*bapA* and complemented strains.

Double-joint PCR was used to generate the deletion constructs of A. nidulans *bapA* (AN1884) and A. flavus *bapA* (AFLA_036020) (33). Briefly, the deletion construct containing the A. fumigatus *pyrG* marker with 5′ and 3′ flanking regions of *bapA* was introduced into the recipient strain RMJP1.59 (A. nidulans) or NRRL 3357.5 (A. flavus). Three independent Δ*bapA* strains each in A. nidulans (TMK6-1, -35, and -47) and A. flavus (TEO1 2, 8, and 9) were confirmed and analyzed. To generate complemented strains of the Δ*bapA* mutant in A. nidulans, a *bapA*^+^ gene region, including its upstream 2-kb region, was introduced to pHS13 (44) and introduced into E. coli DH5α for transformation. Upon sequence verification of the insert, the purified plasmid was introduced into the recipient Δ*bapA* A. nidulans strain (TMK6). Three independent complemented strains (C′*bapA* 12, 16, and 17) were verified and analyzed.

### Microsome isolation and BaP metabolic activity.

Cells were prepared as described without BaP treatment to capture peak *bapA* mRNA levels (Fig. S2A and B). After 1 day of incubation, cells were filtered, washed, squeeze-dried, and flash frozen in liquid N_2_. Frozen tissue was ground to a fine powder in liquid N_2_ with the addition of glass beads in a mortar and pestle. The powder was resuspended in 30 ml homogenization buffer (0.1 M KPO_4_ at pH 7.25, 0.1 M KCl, 10 mM EDTA at pH 8, 0.25 mM phenylmethylsulfonyl fluoride [PMSF], 0.1 mM dithiothreitol [DTT]) and kept in ice. A sonication probe (1/8 in.) was used on full power for 30 s to homogenize cells and form microsomal structures. Large debris was filtered using Miracloth, and the supernatant was centrifuged for 20 min at 20,000 × *g*. The supernatant was then transferred and centrifuged for 60 min at 105,000 × *g*. The resulting supernatant was discarded, and the pellets were resuspended in 200 μl dilution buffer (0.25 M KPO_4_ at pH 7.25, 20% [vol/vol] glycerol, 10 mM EDTA at pH 8.0, 0.25 mM PMSF, 0.1 mM DTT), flash frozen in liquid N_2_, and stored at −80°C for a maximum of 1 week. The protein concentration was determined with the Coomassie protein assay (Thermo Fisher). BaP metabolism was measured by incubating 2 mg/ml microsomal protein, 1 μM BaP, and 1 μM NADPH in up to 1 ml 50 mM phosphate buffer at pH 7.5 at 37°C for 1 h. As a control, protein was denatured by boiling for 20 min prior to incubation. Metabolites were extracted by adding 2 ml of 2:1 acetone-ethyl acetate and vortexing for 2 min. Solvent was removed and centrifuged at 13,000 × *g* for 10 min and dried under N_2_. Samples were resuspended in 50 μl 1:1 HPLC solvent A-solvent B and analyzed by HPLC.

### Statistics.

Statistical significance was determined using Student's *t* test with a two-tailed distribution and two-sample unequal variance.

### Data availability.

All RNA-seq data files are available from the NCBI Gene Expression Omnibus database (45) under accession no. GSE116804.
