# Supplementary figures and images for: Cytochrome P450 Monooxygenase-Mediated Metabolic Utilization of Benzo[a]Pyrene by Aspergillus Species
Source: mBio. 2019 May 28;10(3):e00558-19. doi: 10.1128/mBio.00558-19 (PMC6538779; doi:10.1128/mBio.00558-19)

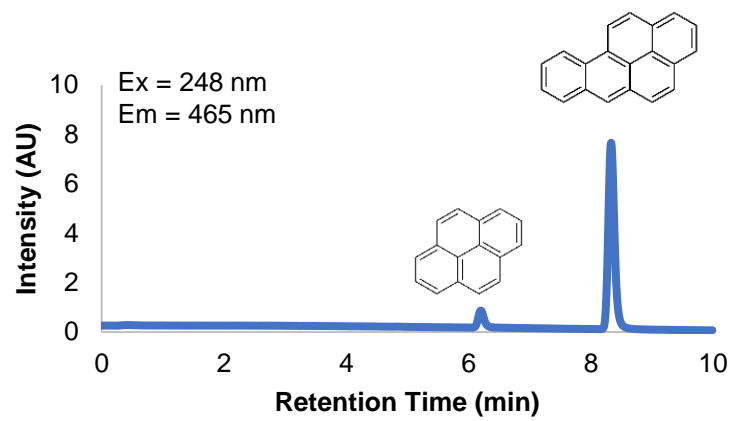

Supplement: FIG S1 [file mBio.00558-19-sf001.pdf]

**A**

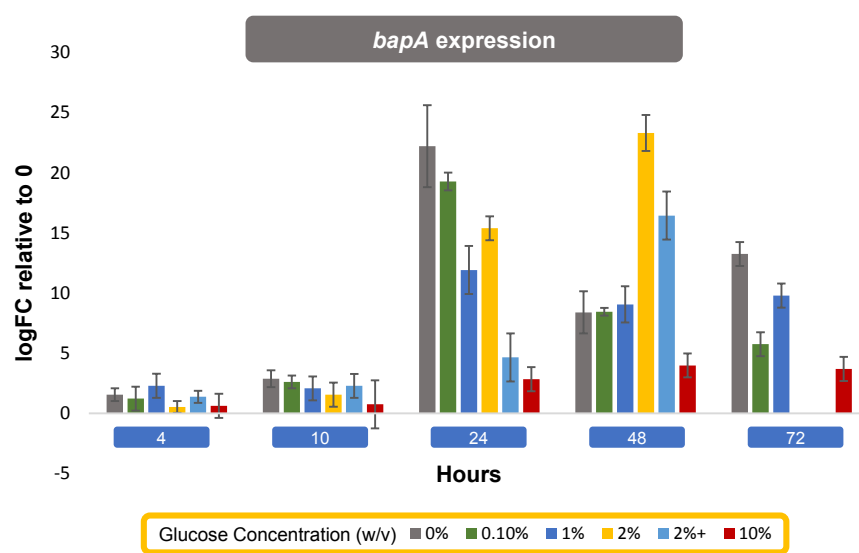

**B**

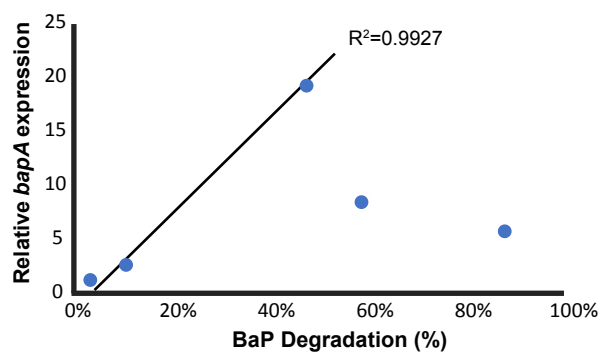

Supplement: FIG S2 [file mBio.00558-19-sf002.pdf]

**A**

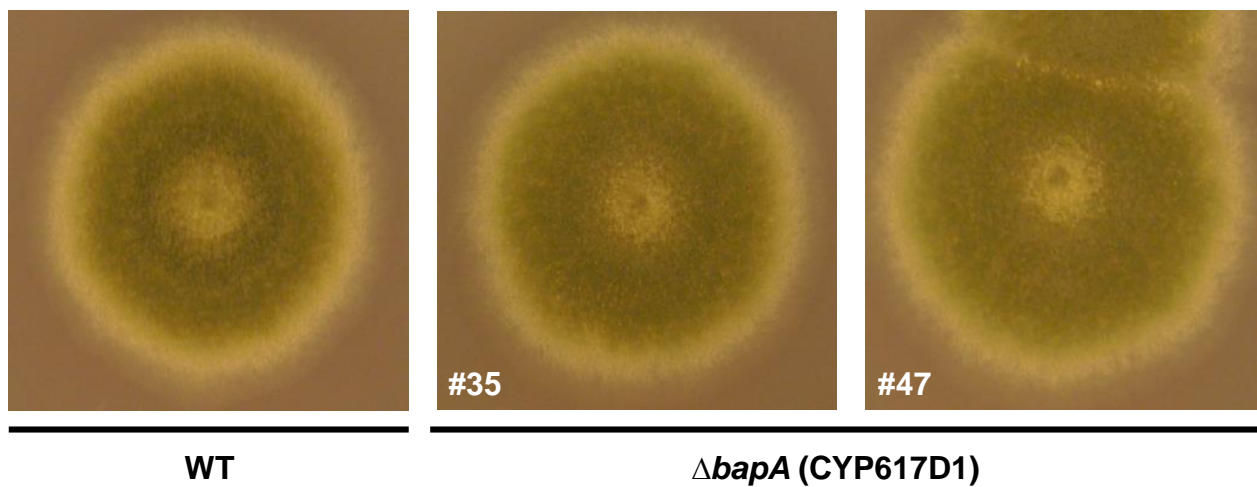

**B**

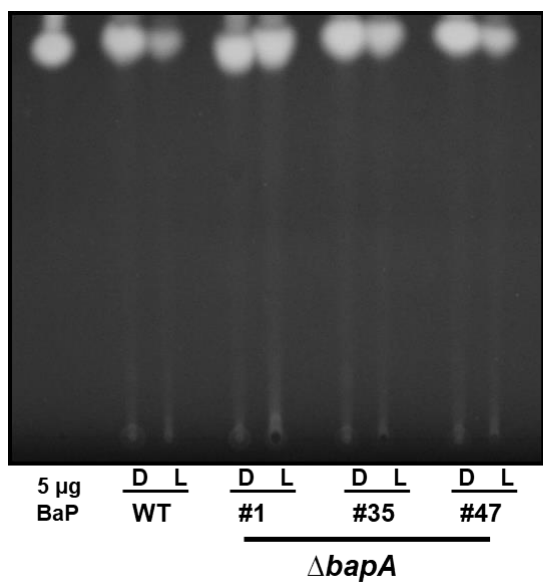

**C**

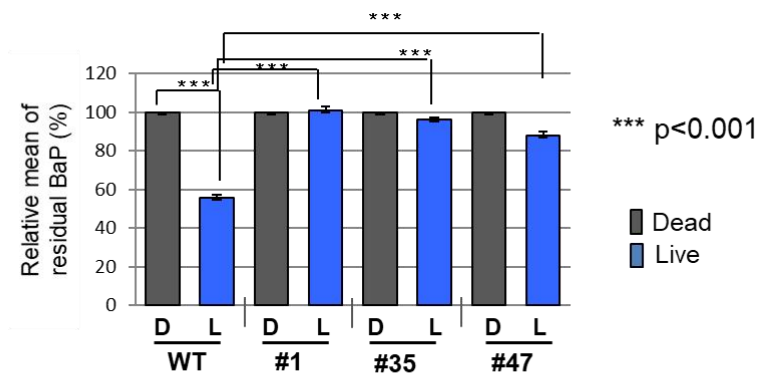

Supplement: FIG S3 [file mBio.00558-19-sf003.pdf]

**A****HPLC chromatogram of microsomes, ex=248 nm, em=465 nm**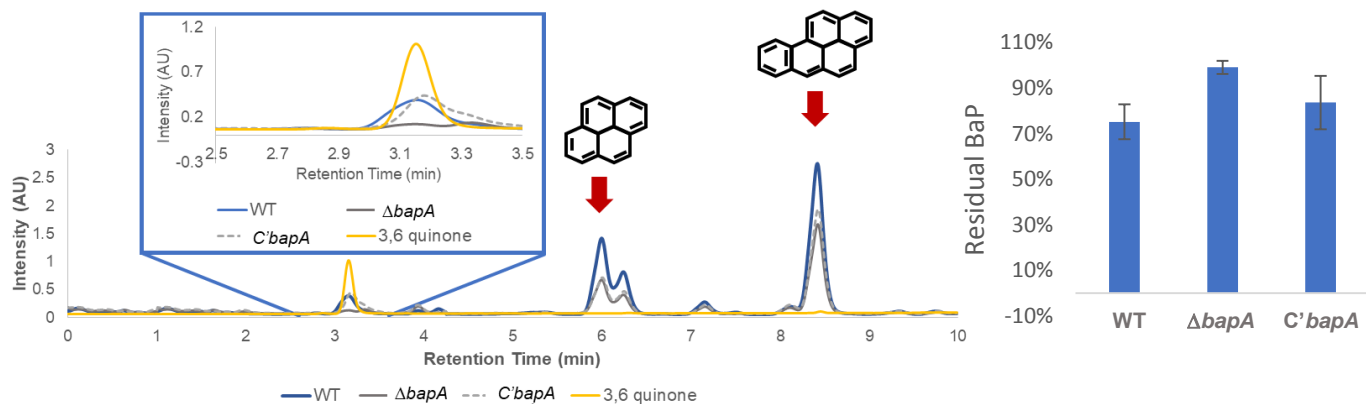**B****Fluorescence Spectra, ex=248 nm**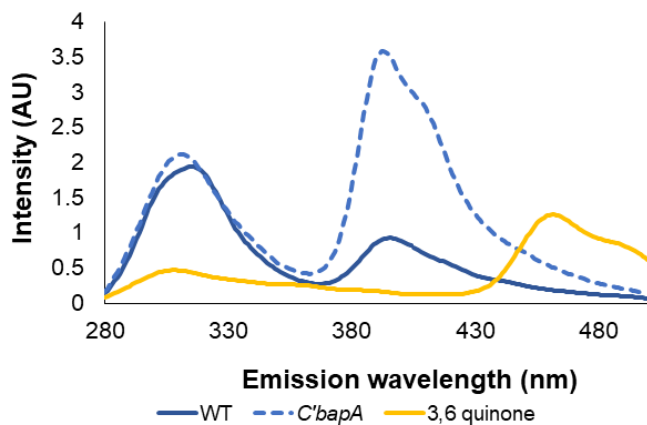**C****1  $\mu$ M standards**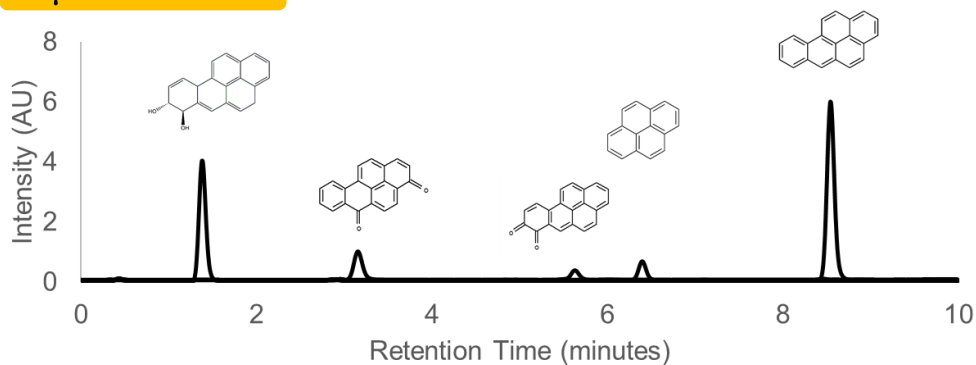

Supplement: FIG S4 [file mBio.00558-19-sf004.pdf]

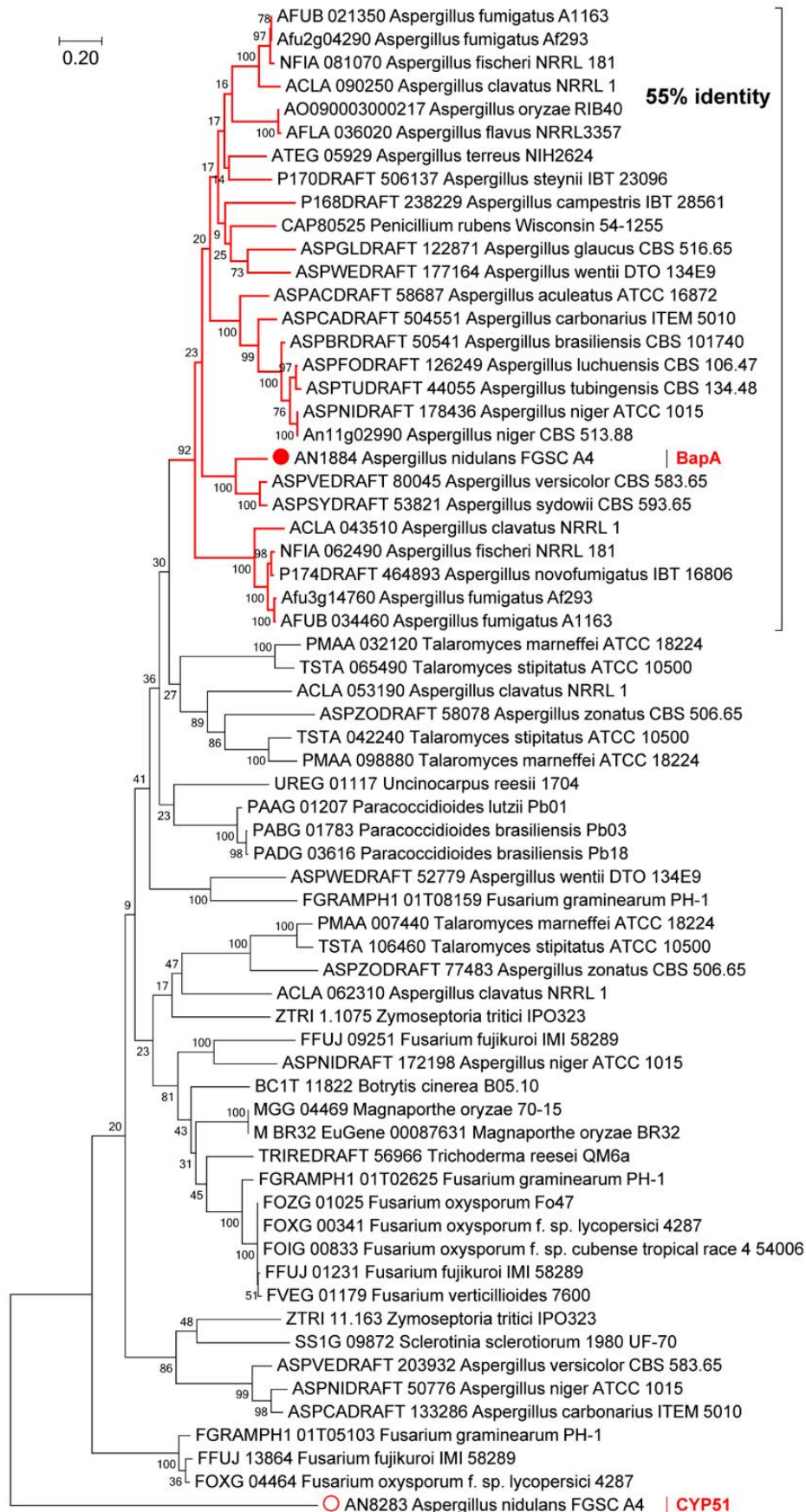

Supplement: FIG S5 [file mBio.00558-19-sf005.pdf]
