# Supplementary material for: Cytochrome P450 Monooxygenase-Mediated Metabolic Utilization of Benzo[a]Pyrene by Aspergillus Species
Source: mBio. 2019 May 28;10(3):e00558-19. doi: 10.1128/mBio.00558-19 (PMC6538779; doi:10.1128/mBio.00558-19)
Supplement: TABLE S1 [file mBio.00558-19-st001.docx]

**Table S1. Oligonucleotides used in this study.**

| **Name** | **Sequence (5' - 3') ^a^** | **Purpose** |
| --- | --- | --- |
| oMK-299 | CGTCGTCATATCACCCTTTG | *bapA*-qPCR 5' |
| oMK-300 | TTCGAATAGACTCGGGCTTT | *bapA*-qPCR 3' |
| oMK-331 | GGCAACATCGTTATGTCTGG | *Aniactin*- qPCR 5' |
| oMK-332 | CCGATCCAGACGGAGTATTT | *Aniactin* qPCR 3' |
| oEO132 | **CAGCTG**GGACTTCGCCGCCATCAA | Complementing △bapA 2kb F' with PvuII |
| oEO133 | **GGATCC**TTACCACCCATCCAATGGCGTC | Complementing △bapA R' with BamHI |
| oMK-55 | TGACATCTTCTTGACTGCCAACG | *AnibapA* flanking region 5' |
| oMK-58 | ATATGCCTCCAGCAGCTACCG | *AnibapA* flanking region 3' |
| oMK-56 | *GCTTTGGCCTGTATCATGACTTCA*GTTGAGATTGGGCCTCGACGAAG | *AnibapA* 3' with *AfupyrG* tail |
| oMK-57 | *ATCGACCGAACCTAGGTAGGGTA*TCTCACTCTGTGTCCATCGAAC | *AnibapA* 5' with *AfupyrG* tail |
| oMK-59 | GAGAACCGTTTCATCGGTATC | *AnibapA* flanking region 5' nested |
| oMK-60 | TCGCCAGTAACTGGCGCAAAC | *AnibapA* flanking region 3' nested |
| oEO1 | ACCCAGAATTTATTTACGCGGAG | *AflbapA* flanking region 5' |
| oEO2 | CATGCATTCGCAGCTTGTGGC | *AflbapA* flanking region 3' |
| oEO3 | *TTTGGCCTGTATCATGACTTCAGC*TCGTGAGCAGAGGCTGCGG | *AflbapA* 3' with *AfupyrG* tail |
| oEO4 | *ATCGACCGAACCTAGGTAGGGTA*ATTGCCCCCGAAATTATCGGTAA | *AflbapA* 5' with *AfupyrG* tail |
| oEO5 | CGGAGCGGTTCAAGAGA | *AflbapA* flanking region 5' nested |
| eEO6 | GGGGACTGGAGTTCATGGATG | *AflbapA* flanking region 3' nested |
| oEO-62 | TTCGAGATGGAGCTGGCA | Plasmid sequence promoter region 5’ |
| oEO-70 | CTATTACTTGTCATCGTCATCCT | Plasmid sequence 3x flag 3’ |

**^a^** Tail sequences are shown in italics. Restriction enzyme sites are in bold.
