# Supplementary material for: Cytochrome P450 Monooxygenase-Mediated Metabolic Utilization of Benzo[a]Pyrene by Aspergillus Species
Source: mBio. 2019 May 28;10(3):e00558-19. doi: 10.1128/mBio.00558-19 (PMC6538779; doi:10.1128/mBio.00558-19)
Supplement: TABLE S2 [file mBio.00558-19-st002.docx]

**Table S2. CYPs similar to Pc-PAH1 and Pc-PAH3 in *A. nidulans*, *A. flavus,* and *A. fumigatus.***

| PC-Pah | **CYP Calss (aa#)** | ***A. nidulans***  **locus** | **Identity** | **E-value** | ***A. flavus***  **locus** | **E-value** | ***A. fumigatus***  **locus** | **E-value** |
| --- | --- | --- | --- | --- | --- | --- | --- | --- |
| **Pc-PAH1** | CYP617D1 (544) | **AN1884** | 32% | 3e-66 | AFL2G_02761 | 4.00E-99 | Afu2g04290 | 0.00E+00 |
|  | CYP547C1 (549) | AN11142 | 27% | 4E-23 | AFL2G_11890 | 0.00E+00 | Afu8g02610 | 0.00E+00 |
|  | CYP539D1 (538) | AN3917 | 24% | 8E-21 | AFL2G_09153 | 0.00E+00 | Afu6g08460 | 0.00E+00 |
|  | CYP680A1 (515) | AN0338 | 25% | 4e-12 | AFL2G_11819 | 5.00E-75 | Afu5g01360 | 6.00E-73 |
| **Pc-PAH3** | CYP620E1 (524) | AN1601 | 36% | 1e-99 | AFL2G_00089 | 0.00E+00 | Afu8g00510 | 7.00E-124 |
|  | CYP663A1 (543) | AN7399 | 31% | 2e-59 | AFL2G_02173 | 2.00E-64 | Afu1g11390 | 4.00E-69 |
|  | CYP677A1 (502) | AN8615 | 31% | 2e-08 | AFL2G_06876 | 2.00E-76 | Afu5g01360 | 3.00E-50 |
